# Correlation analysis between myocardial work indices and liver function classification in patients with hepatitis B cirrhosis: A study with non-invasive left ventricular pressure-strain loop

**DOI:** 10.3389/fcvm.2023.1126590

**Published:** 2023-03-08

**Authors:** Yang Cao, Huihui Zhang, Shuai Li, Siliang Li, Shuowen Sun, Jinwen Chen, Ting Ye, Xijun Zhang, Jianjun Yuan

**Affiliations:** ^1^ Department of Ultrasound, People’s Hospital of Zhengzhou University, Henan Provincial People’s Hospital, Zhengzhou, China; ^2^ Department of Ultrasound, Henan Provincial People’s Hospital, Zhengzhou, China; ^3^ Department of Ultrasound, People’s Hospital of Henan University, Henan Provincial People’s Hospital, Zhengzhou, China

**Keywords:** liver cirrhosis, myocardial work, pressure-strain loop, echocardiography, left ventricular

## Abstract

**Background:**

Liver cirrhosis is closely associated with cardiac dysfunction. The aims of this study were to evaluate left ventricular systolic function in patients with hepatitis B cirrhosis by non-invasive left ventricular pressure-strain loop (LVPSL) technique, and to explore the correlation between myocardial work indices and liver function classification.

**Methods:**

According to the Child-Pugh classification, 90 patients with hepatitis B cirrhosis were further divided into three groups: Child-Pugh A group (*n* = 32), Child-Pugh B group (*n* = 31), and Child-Pugh C group (*n* = 27). During the same period, 30 healthy volunteers were recruited as the control (CON) group. Myocardial work parameters, which included global work index (GWI), global constructive work (GCW), global wasted work (GWW), and global work efficiency (GWE), were derived from the LVPSL and compared among the four groups. The correlation between myocardial work parameters and Child-Pugh liver function classification was evaluated, and the independent risk factors affecting left ventricular myocardial work in patients with cirrhosis were investigated by univariable and multivariable linear regression analysis.

**Results:**

GWI, GCW and GWE of Child-Pugh B and C groups were lower than those of CON group, while GWW was higher than that of CON group, and the changes were more obvious in Child-Pugh C group (*P* < 0.05). Correlation analysis revealed that GWI, GCW, and GWE were negatively correlated with liver function classification to various degrees (*r* = −0.54, −0.57, and −0.83, respectively, all *P* < 0.001), while GWW was positively correlated with liver function classification (*r* = 0.76, *P* < 0.001). Multivariable linear regression analysis showed that GWE was positively correlated with ALB (*β* = 0.17, *P* < 0.001), and negatively correlated with GLS (*β* = −0.24, *P* < 0.001).

**Conclusions:**

The changes in the left ventricular systolic function in patients with hepatitis B cirrhosis were identified using non-invasive LVPSL technology, and myocardial work parameters are significantly correlated with liver function classification. This technique may provide a new method for the evaluation of cardiac function in patients with cirrhosis.

## 1. Introduction

Liver cirrhosis is a common chronic progressive liver disease with a high mortality rate, which may cause multiple system dysfunction in the advanced stage. In Asian populations, chronic viral hepatitis B is the primary cause of liver cirrhosis (1). Cirrhotic cardiomyopathy (CCM) is one of the significant complications of cirrhosis and is closely related to the poor prognosis of patients with liver cirrhosis (2). It comprises a triad of impaired myocardial contractile responses to stress (systolic dysfunction), inadequate ventricular relaxation (diastolic dysfunction), and electrophysiological abnormalities in the absence of any known cardiac disease (3). Patients with cirrhosis may have various cardiovascular complications and even induce heart failure under stress conditions such as load or clinical invasive operation (4, 5). Heart failure due to CCM is claimed to the third cause of mortality in liver transplant patients following infection and rejection (6). Therefore, the evaluation of left ventricular systolic function in patients with cirrhosis in the early stage is extremely important for treatment and prognosis.

Left ventricular systolic function and myocardial oxygen consumption can be accurately assessed with the use of pressure-volume loop measured by cardiac catheterization, but this method is an invasive examination with limited use in clinical practice (7). The left ventricular global longitudinal strain (GLS) measured by two-dimensional speckle tracking echocardiography (2D-STE) can be used to quantitatively evaluate left ventricular systolic function, but its load-dependent limitations affect the objective evaluation of myocardial systolic function (8). The left ventricular pressure-strain loop (LVPSL) is a new technique developed on the basis of 2D-STE, which allows for a more accurate assessment of left ventricular myocardial work by considering the effect of afterload on strain (9, 10). The results of non-invasive LVPSL in assessing myocardial work were significantly correlated with the invasive cardiac catheterization results (11). This method is simple, non-invasive, and reproducible, allowing for a more objective and accurate assessment of left ventricular function.

LVPSL technology has been widely used in the diagnosis of many cardiovascular diseases, but no studies have yet applied it to patients with cirrhosis. We hypothesized that LVPSL might provide incremental value for the assessment of left ventricular systolic function in patients with cirrhosis. The aim of this study was (1) to evaluate the left ventricular myocardial work of patients with different degrees of hepatitis B cirrhosis by using LVPSL technology; (2) to explore the correlation between myocardial work indices and liver function classification; (3) to find the clinical factors impairing the left ventricular myocardial work.

## 2. Materials and methods

### 2.1. Study population

**Figure 1:**
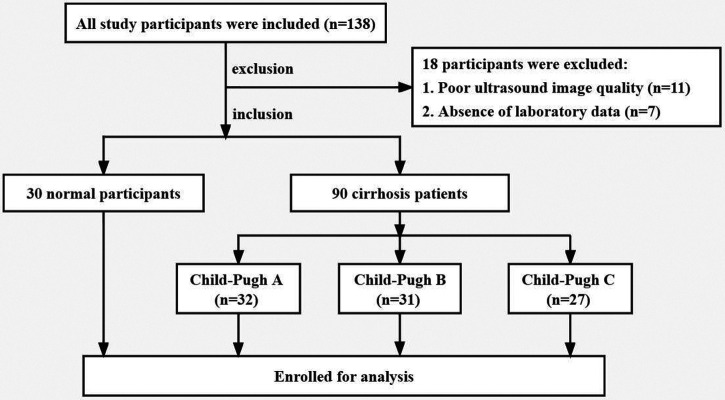
Flowchart of study populations.

From July 2020 to May 2021, a total of 90 patients with hepatitis B cirrhosis were recruited in this study, including 50 males and 40 females. The diagnosis of liver cirrhosis is based on clinical symptoms, laboratory data, imaging and pathological examination (12). According to the Child-Pugh classification, the patients with liver cirrhosis were divided into three groups: Child-Pugh A group (*n* = 32, 56% men, age 42.9 ± 9.4 years), Child-Pugh B group (*n* = 31, 55% men, age 41.8 ± 7.6 years), and Child-Pugh C group (*n* = 27, 56% men, age 41.5 ± 10.6 years). Thirty healthy volunteers were allocated to the control (CON) group (57% men, age 41.9 ± 10.9 years). The exclusion criteria were as follows: (i) coronary atherosclerotic heart disease, hypertension, congenital heart disease and other cardiovascular diseases; (ii) patients with chronic kidney disease, chronic respiratory disease, thyroid dysfunction and other diseases that may lead to secondary heart damage; (iii) patients with gastrointestinal bleeding in the last month; (iv) diabetes, hyperlipidemia and obesity; (v) poor-quality of ultrasound images. The participant selection process is illustrated in the flowchart in Figure 1. This study was approved by the ethics committee of Henan Provincial People's Hospital and informed consent was obtained from all subjects before image acquisitions.

### 2.2. Clinical features

Clinical data of all subjects were collected, such as age, BMI, BSA, heart rate (HR), systolic blood pressure (SBP), diastolic blood pressure (DBP) and pulse pressure (PP). Albumin (ALB), total bilirubin (TBil), alanine aminotransferase (ALT), aspartate aminotransferase (AST), and brain natriuretic peptide (BNP) level in patients with cirrhosis were obtained by standard laboratory techniques.

### 2.3. Conventional ultrasonic parameters

All subjects were underwent conventional echocardiography examination according to the American Society of Echocardiography guidelines (13), using a GE Vivid E95 colour Doppler ultrasound system (GE Vingmed Ultrasound AS, Horten, Norway) equipped with M5Sc-D transducer (1.4–4.6 MHz) and C1-6 transducer (3.5–5.0 MHz). Before examination, the brachial artery blood pressure was measured three times with an electronic manometer, and then the average was taken for analysis (assuming left ventricular systolic pressure equal to the brachial artery pressure). All subjects were instructed to take a supine position and breathe calmly. Using the C1-6 probe, the portal vein inner diameter (Dpv) and its flow velocity (Vpv) were obtained in the first longitudinal section of the hepatic portal under the right costal margin. Then the subjects were instructed to assume the left-lateral position and the electrocardiogram was attached. By adjusting the frequency, gain and image size, the endocardial surface was clearly displayed. Conventional parameters were measured in the parasternal long-axis view of the left ventricle, such as the left atrial diameter (LAD), left ventricular end-diastolic diameter (LVDd), left ventricular end-systolic diameter (LVDs), interventricular septum thickness (IVST) and left ventricular posterior wall thickness (LVPWT). Left ventricular ejection fraction (LVEF) was calculated by Simpson's biplane method. The mitral and aortic valve Doppler spectral images were obtained. Dynamic images consisting of five consecutive cardiac cycles from the apical four-, three-, and two-chamber views were collected at frame rate of 50–70 frames/s, and the images were stored and copied to a mobile hard disk for analysis.

### 2.4. Quantitative analysis of GLS and myocardial work parameters

**Figure 2:**
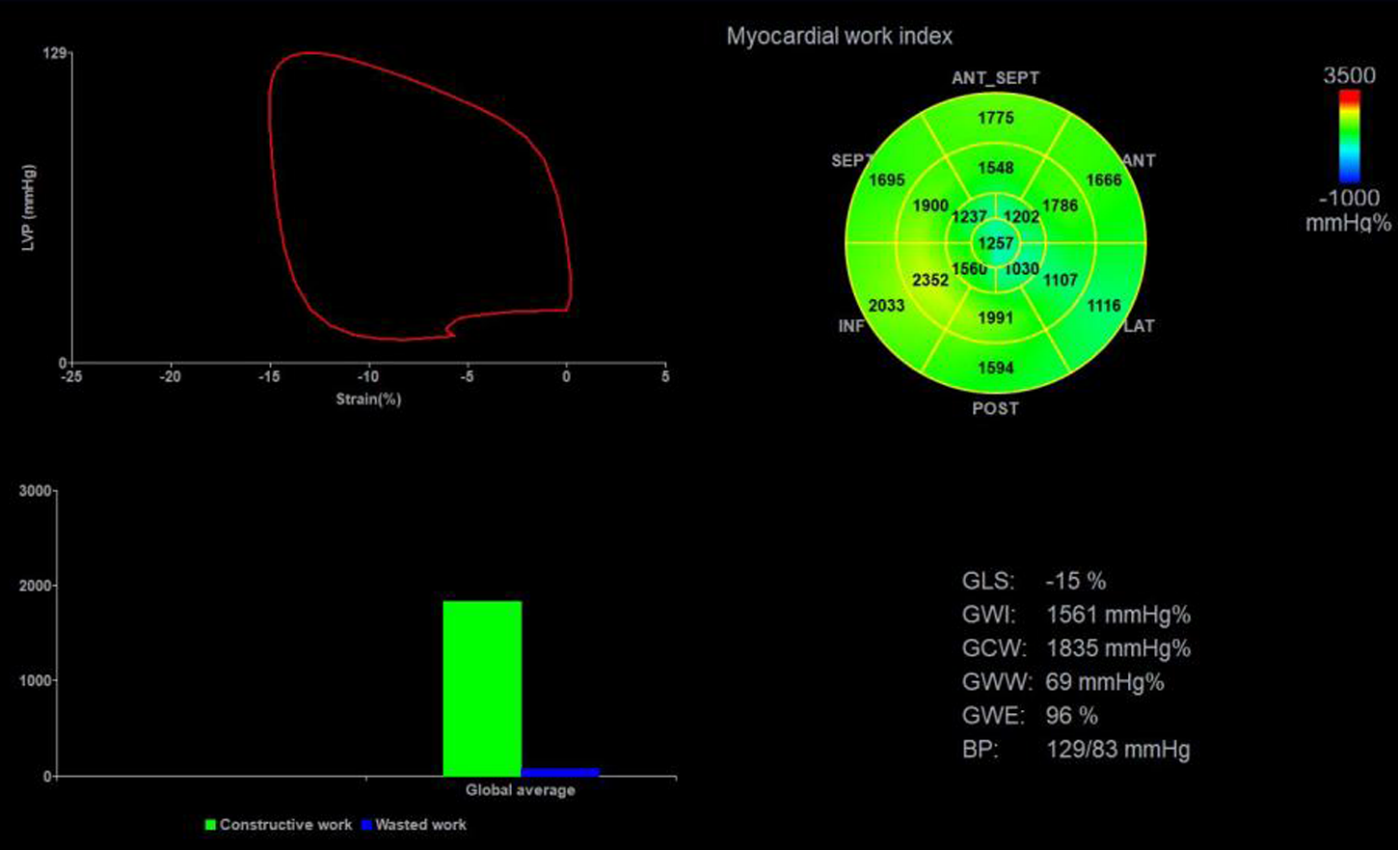
Left ventricular myocardial work parameters were obtained by non-invasive PSL technique. Top left: PSL curve; Top right: the 17-segment myocardial work index bull's eye diagram; Bottom left: comparison diagram of GCW (green column) and GWW (blue column); Bottom right: the parameters regarding myocardial work. PSL, pressure-strain loop; GLS, global longitudinal strain; GWI, global work index; GWE, global work efficiency; GCW, global constructive work; GWW, global wasted work.

The stored dynamic images were imported into the Echopac version 203 workstation (GE vingmed ultrasound, Horten, Norway) in original format for offline analysis. The aortic valve closure time was marked by the anterior flow spectrogram of the aortic valve to define the duration of isovolumic contraction, ejection, and isovolumic diastolic. The software can automatically identify and track the left ventricular myocardial motion trajectory and the region of interest was adjusted by correcting the endocardial border or width until a satisfactory image was obtained. Finally, after entering the mean brachial artery blood pressure value, the software automatically obtained GLS, the LVPSL curve, and myocardial work parameters, as shown in Figure 2. The myocardial work parameters are as follows:

(1) Global work index (GWI): the total work within the area of the LVPSL curve calculated from mitral valve closure to mitral valve opening.
(2) Global constructive work (GCW): work performed by shortening in systole and lengthening during the isovolumic diastole phase.
(3) Global wasted work (GWW): the negative work performed by lengthening in systole and shortening during the isolvolumic diastole phase.
(4) Global work efficiency (GWE): the percentage of GCW in the sum of GCW and GWW.

### 2.5. Statistical analysis

All statistical analyses were performed using SPSS version 26.0 software (IBM, Armonk, NY, USA). Continuous variables with normal distribution were presented as mean ± standard deviation, and those with non-normal distribution were presented as median (interquartile range). One-way analysis of variance was used for comparison among multiple groups when the variances were homogeneous, and the least significant difference t-test was used for further pairwise comparison. The rank sum test was used to compare non-normally distributed data. Categorical variables were expressed as frequencies and percentages and compared by the *x*^2^-test between the groups. Correlations between myocardial work parameters and Child-Pugh liver function classification were examined by Spearman correlation coefficients. The factors with *P*-value <0.05 in univariable linear regression results were incorporated into the multivariable linear regression analysis models by means of stepwise selection to detect the independent predictors of abnormal myocardial function in patients with liver cirrhosis. In the multivariable linear regression model, there was no multicollinearity between variables. Intra-observer and inter-observer variability of myocardial work parameters were assessed in 15 cirrhosis patients and 15 healthy volunteers selected randomly and tested using the intraclass correlation coefficients (ICCs). All tests were two-sided, and *P* < 0.05 was considered statistically significant.

## 3. Results

### 3.1. Clinical and laboratory parameters

A total of 90 patients with liver cirrhosis were enrolled in this study, with an average age of 42.1 ± 9.1 years. The clinical and laboratory characteristics of the study subjects are illustrated in Table 1. Compared with the CON group, TBil, ALT, AST and BNP were significantly increased, and ALB was decreased in the liver cirrhosis groups, especially in the Child-Pugh C group (*P *< 0.05). There were no significant differences in sex, age, BMI, BSA, HR, SBP, DBP, and PP among the four groups (*P *> 0.05).

**Table 1:** Clinical and laboratory parameters.

	CON (*n* = 30)	Child-Pugh A (*n* = 32)	Child-Pugh B (*n* = 31)	Child-Pugh C (*n* = 27)	*P*-value
Male gender, *n* (%)	17 (57%)	18 (56%)	17 (55%)	15 (56%)	0.99
Age (years)	42.0 ± 10.9	42.9 ± 9.4	41.8 ± 7.6	41.5 ± 10.6	0.95
BMI (kg/m²)	23.4 ± 2.7	23.2 ± 2.9	23.2 ± 2.3	23.3 ± 3.0	0.99
BSA (m²)	1.7 ± 0.3	1.7 ± 0.4	1.7 ± 0.4	1.7 ± 0.4	0.99
HR (bpm)	68.8 ± 9.6	70.4 ± 9.6	71.7 ± 10.4	71.5 ± 6.6	0.62
SBP (mm Hg)	124.6 ± 9.2	121.8 ± 11.8	119.4 ± 9.4	119.9 ± 11.3	0.21
DBP (mm Hg)	77.0 ± 8.8	76.8 ± 8.8	75.2 ± 5.5	75.3 ± 7.1	0.71
PP (mm Hg)	47.6 ± 9.0	45.0 ± 10.7	44.2 ± 8.7	44.6 ± 9.9	0.52
ALB (g/L)	43.7 ± 6.7	39.4 ± 5.3*	32.0 ± 8.4*^,^**	22.9 ± 6.1*^,^**^,^***	<0.001
TBil (µmol/L)	12.1 ± 3.1	26.0 ± 6.8*	49.7 ± 10.9*^,^**	66.6 ± 14.2*^,^**^,^***	<0.001
ALT (U/L)	24.6 ± 6.4	36.4 ± 4.6*	52.1 ± 9.9*^,^**	69.0 ± 12.4*^,^**^,^***	<0.001
AST (U/L)	23.9 ± 6.1	33.8 ± 7.7*	52.9 ± 14.3*^,^**	81.4 ± 11.9*^,^**^,^***	<0.001
BNP (pg/ml)	34.5 ± 9.0	68.7 ± 10.3*	128.5 ± 14.5*^,^**	186.8 ± 42.3*^,^**^,^***	<0.001

Data are expressed as mean ± SD or as number (percentage).

BMI, body mass index; BSA, body surface area; HR, heart rate; SBP, systolic blood pressure; DBP, diastolic blood pressure; PP, pulse pressure; ALB, Albumin; TBil, total bilirubin; ALT, alanine aminotransferase; AST, aspartate aminotransferase; BNP, brain natriuretic peptide.

* *P* < 0.05 vs. CON; **P < 0.05 vs. Child-Pugh A; ^,^***P < 0.05 vs. Child-Pugh B.

### 3.2. Conventional ultrasound parameters

The conventional ultrasound parameters of each group are summarized in Table 2. LAD, IVST and LVPWT in the cirrhosis groups were increased compared to the CON group (*P *< 0.05). Compared with the CON group and the Child-Pugh A group, Child-Pugh B and C groups had higher Dpv and lower Vpv (*P *< 0.05). No significant differences were identified among the four groups in terms of LVDd, LVDs and LVEF (*P *> 0.05).

**Table 2:** Conventional ultrasound parameters.

	CON (*n* = 30)	Child-Pugh A (*n* = 32)	Child-Pugh B (*n* = 31)	Child-Pugh C (*n* = 27)	*P*-value
Dpv (mm)	9.9 ± 1.4	10.6 ± 1.6	12.7 ± 1.6*^,^**	14.3 ± 1.2*^,^**^,^***	<0.001
Vpv (m/s)	19.9 ± 4.1	18.1 ± 3.0	15.8 ± 2.4*^,^**	13.8 ± 2.0*^,^**^,^***	<0.001
LAD (mm)	30.7 ± 2.9	33.9 ± 2.6*	35.1 ± 4.7*	39.0 ± 4.0*^,^**^,^***	<0.001
LVDd (mm)	47.1 ± 3.4	48.2 ± 4.3	47.8 ± 3.4	48.5 ± 3.4	0.52
LVDs (mm)	27.8 ± 3.9	28.8 ± 3.8	28.3 ± 4.0	29.1 ± 3.8	0.63
IVST (mm)	8.4 ± 1.2	9.9 ± 1.2*	10.1 ± 1.5*	10.3 ± 1.5*	<0.001
LVPWT (mm)	8.6 ± 0.9	9.6 ± 1.2*	10.0 ± 1.4*	10.2 ± 1.4*	<0.001
LVEF (%)	67.5 ± 5.2	68.1 ± 5.0	69.6 ± 6.8	68.6 ± 5.9	0.56

Data are expressed as mean ± SD. Dpv, portal vein inner diameter; Vpv, portal vein flow velocity; LAD, left atrial diameter; LVDd, left ventricular end-diastolic diameter; LVDs, left ventricular end-systolic diameter; IVST, interventricular septum thickness; LVPWT, left ventricular posterior wall thickness; LVEF, left ventricular ejection fraction. *P < 0.05 vs. CON; ^,^**P < 0.05 vs. Child-Pugh A; ^,^***P < 0.05 vs. Child-Pugh B.

### 3.3. GLS and myocardial work parameters

**Figure 3:**
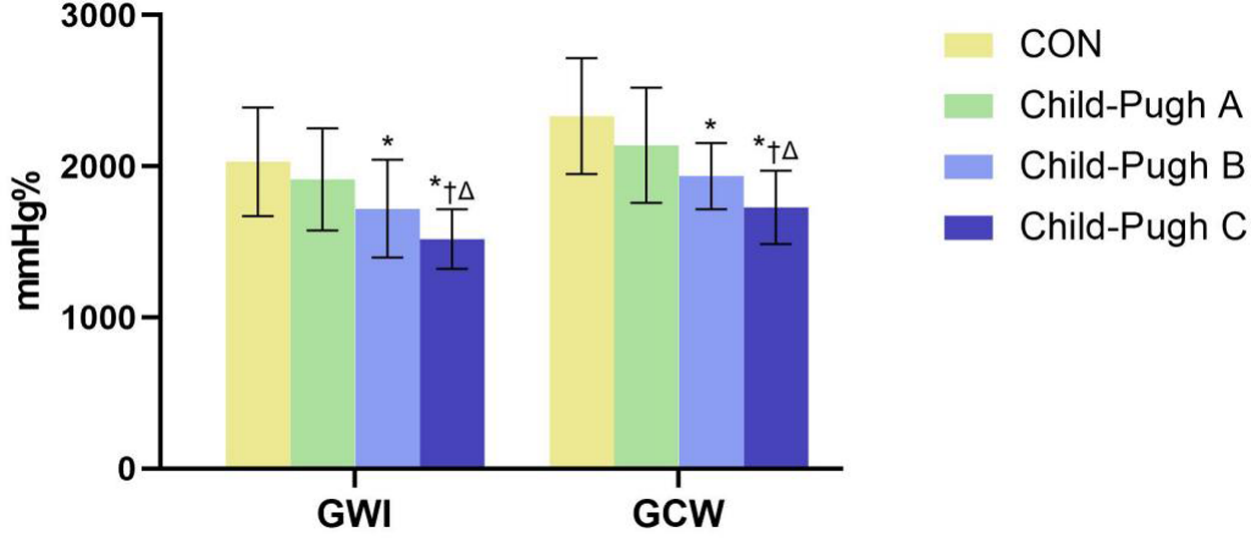
The bar chart showed differential changes of GWI and GCW among the four groups. GWI, global work index; GCW, global constructive work. *P < 0.05 vs. CON. †*P* < 0.05 vs. Child-Pugh A. ΔP < 0.05 vs. Child-Pugh B.

**Figure 4:**
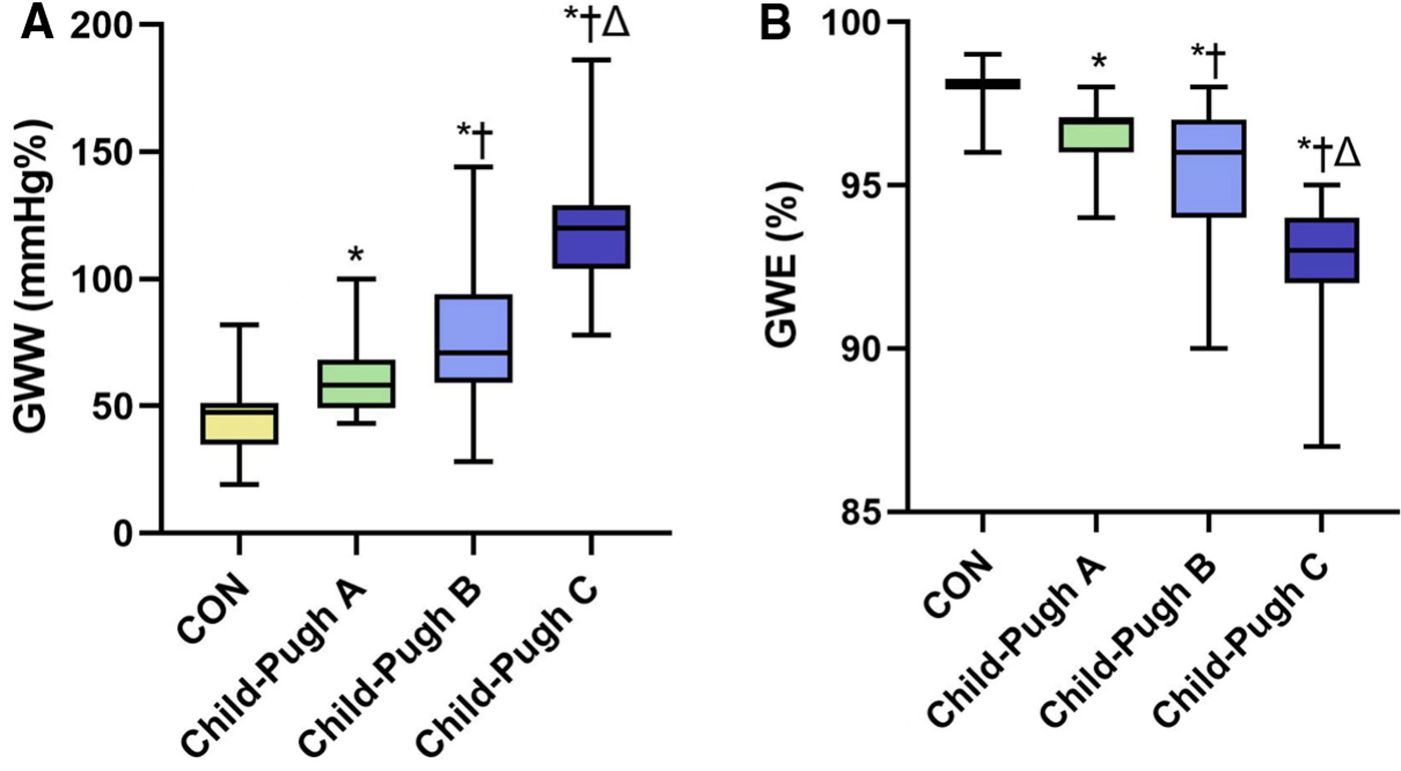
The boxplots showed differential changes of GWW (**A**) and GWE (**B**) among the four groups. GWW, global wasted work; GWE, global work efficiency. *P < 0.05 vs. CON. †*P* < 0.05 vs. Child-Pugh A. ΔP < 0.05 vs. Child-Pugh B.

The absolute value of GLS in the cirrhosis groups was significantly decreased compared to the CON group, especially in the Child-Pugh C group (*P *< 0.05). Compared with the CON group, GWI, GCW and GWE in Child-Pugh B and C groups were significantly decreased, while GWW was increased (*P *< 0.05). Compared with Child-Pugh A and B groups, GWI, GCW and GWE in Child-Pugh C group were further decreased, while GWW was further increased (*P *< 0.05). (Table 3 and Figures 3, 4).

**Table 3:** GLS and myocardial work parameters.

	CON (*n* = 30)	Child-Pugh A (*n* = 32)	Child-Pugh B (*n* = 31)	Child-Pugh C (*n* = 27)	*P*-value
GLS (%)	−21.2 ± 2.3	−19.4 ± 2.3*	−17.4 ± 3.3*^,^**	−15.0 ± 2.5*^,^**^,^***	<0.001
GWI (mm Hg%)	2,030.2 ± 358.4	1,914.1 ± 337.3	1,719.7 ± 324.3*	1,518.0 ± 198.0*^,^**^,^***	<0.001
GCW (mm Hg%)	2,331.8 ± 384.1	2,140.0 ± 381.6	1,935.6 ± 218.5*	1,728.6 ± 242.2*^,^**^,^***	<0.001
GWW (mm Hg%)	47.5 (16.5)	58.0 (19.3)*	71.0 (35.0)*^,^**	120.0 (25.0)*^,^**^,^***	<0.001
GWE (%)	98.0 (0.3)	97.0 (1.0)*	96.0 (3.0)*^,^**	93.0 (2.0)*^,^**^,^***	<0.001

Data are expressed as mean ± SD or as median (quartile range). GLS, global longitudinal strain; GWI, global work index; GCW, global constructive work; GWW, global wasted work; GWE, global work efficiency.

* *P* < 0.05 vs. CON; ^,^**P < 0.05 vs. Child-Pugh A; ^,^***P < 0.05 vs. Child-Pugh B.

### 3.4. Correlation between myocardial work parameters and Child-Pugh classification

**Figure 5:**
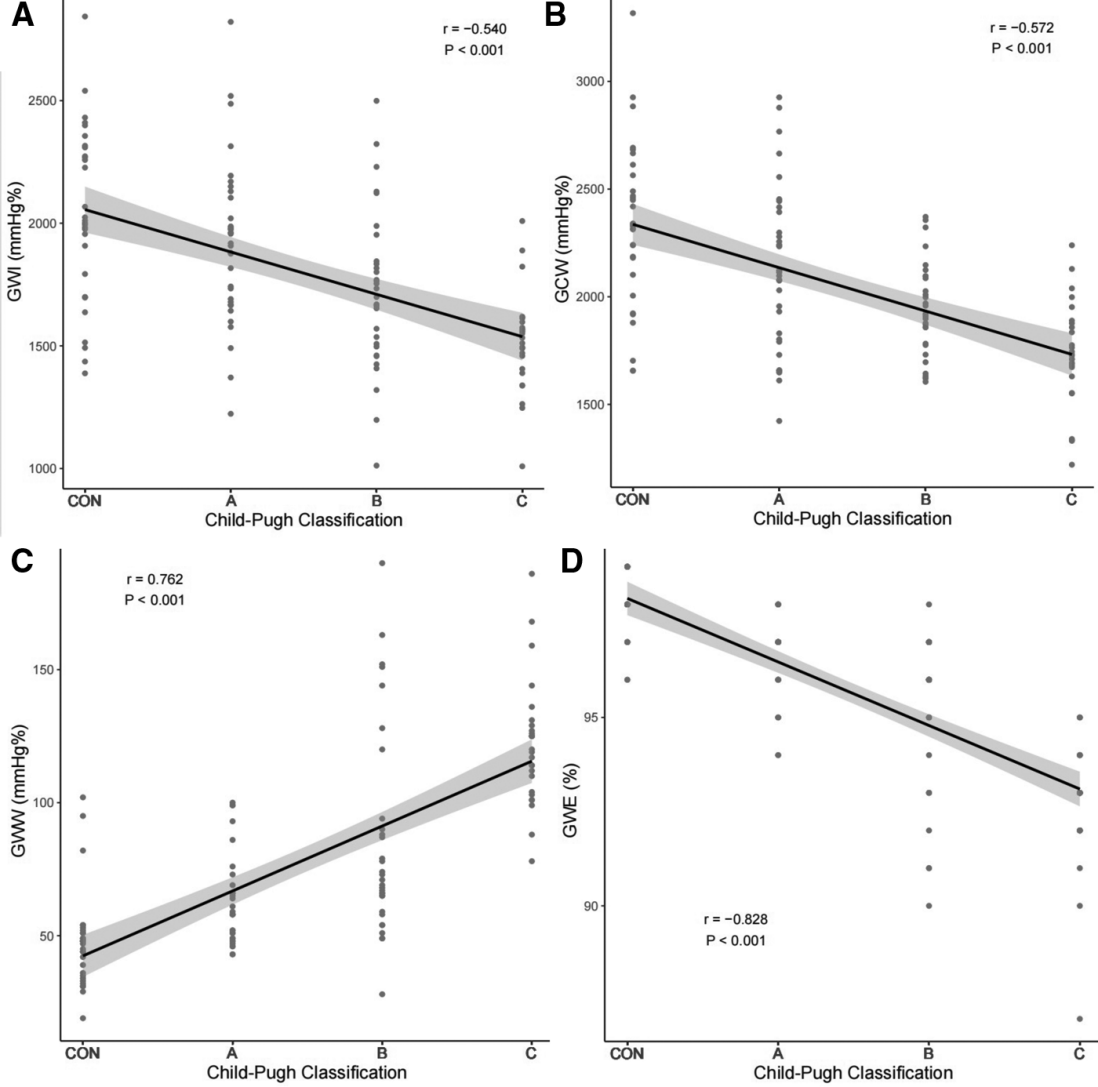
Correlation of GWI (**A**), GCW (**B**), GWW (**C**), GWE (**D**) with Child-Pugh classification. GWI, global work index; GCW, global constructive work; GWW, global wasted work; GWE, global work efficiency.

Spearman correlation analysis results showed that GWI, GCW, GWE were negatively correlated with Child-Pugh classification (GWI: *r* = −0.54, *P* < 0.001; GCW: *r* = −0.57, *P* < 0.001; GWE: *r* = −0.83, *P* < 0.001) and GWW was positively correlated with Child-Pugh classification (GWW: *r* = 0.76, *P* < 0.001). GWE showed more significant correlation than other myocardial work parameters, as exhibited in Figure 5.

### 3.5. Potential associated factors for GWE in cirrhosis patients

HR, ALB, TBil, Dpv, IVST, LVPWT and GLS were incorporated into the multivariable linear regression analysis model of GWE by means of stepwise selection based on the univariable linear regression analysis results. The results showed that GWE was positively correlated with ALB (*β* = 0.17, *P *< 0.001), and negatively correlated with GLS (*β* = −0.24, *P* < 0.001). The detailed data are listed in Table 4.

**Table 4:** Potential associated factors of GWE in cirrhosis patients.

Variables	Univariable analysis		Multivariable analysis	
	*β*	*P*-value	*β*	*P*-value
Age	−0.01	0.57	–	–
BSA	−0.46	0.42	–	–
BMI	0.03	0.76	–	–
HR	−0.05	0.04	–	–
ALB	0.21	<0.001	0.17	<0.001
TBil	−0.05	<0.001	–	–
Dpv	−0.65	<0.001	–	–
IVST	−0.45	<0.001	–	–
LVPWT	−0.44	0.004	–	–
LVEF	−0.05	0.19	–	–
GLS	−0.48	<0.001	−0.24	<0.001

GWE, global work efficiency; BSA, body surface area; BMI, body mass index; HR, heart rate; ALB, albumin; TBil, total bilirubin; Dpv, portal vein inner diameter; IVST, interventricular septum thickness; LVPWT, left ventricular posterior wall thickness; LVEF, left ventricular ejection fraction; GLS, global longitudinal strain.

### 3.6. Reproducibility test

Intra-observer and inter-observer variability for myocardial work parameters are summarized in Table 5. The results showed good repeatability and reproducibility in global myocardial work parameters.

**Table 5:** Reproducibility test.

	Intra-observer variability			Inter-observer variability		
	ICC	95% CI	*P*-value	ICC	95% CI	*P*-value
GWI	0.98	0.96–0.99	<0.001	0.98	0.95–0.99	<0.001
GCW	0.97	0.94–0.99	<0.001	0.96	0.89–0.98	<0.001
GWW	0.97	0.93–0.99	<0.001	0.96	0.93–0.98	<0.001
GWE	0.97	0.94–0.99	<0.001	0.98	0.95–0.99	<0.001

ICC, intraclass correlation coefficient; CI, confidence interval; GWI, global work index; GCW, global constructive work; GWW, global wasted work; GWE, global work efficiency.

## 4. Discussion

This study mainly illustrates the myocardial function of patients with different degrees of hepatitis B cirrhosis by non-invasive PSL technique. The main findings are as follows: (1) Compared with the CON group, there was no significant change in LVEF in the cirrhosis groups, but the myocardial work parameters had changed. GWI, GCW and GWE in Child-Pugh B and C groups were significantly lower than those in control group, while GWW was significantly higher than that in control group, and the change was more obvious in Child-Pugh C group. (2) GWI, GCW and GWE were negatively correlated with Child-Pugh classification, while GWW was positively correlated with Child-Pugh classification. (3) GWE was independently correlated with ALB and GLS respectively.

Liver cirrhosis is the terminal stage of various chronic liver diseases with high mortality rate, which can damage the function of other organs to varying degrees, and it has become a serious public health problem (14). As one of the important complications of cirrhosis, CCM is closely related to the prognosis and survival rate of patients, and has gradually received clinical attention in recent years. The onset of CCM is usually insidious and the myocardial damage is not obvious in the resting state, but it may cause serious adverse consequences during some clinical procedures that affect hemodynamics (15). Therefore, early detection of cardiac function impairment in patients with cirrhosis and evaluation of its relationship with the progression of cirrhosis may be crucial for clinical diagnosis and treatment, prognosis assessment and prevention of cardiovascular adverse events.

Previous studies have shown that GLS can reflect left ventricular systolic function sensitively (16, 17). In this study, the absolute value of GLS in cirrhosis patients was lower than that in the control group. With the deterioration of liver function, the absolute value of GLS further decreased, which was consistent with the results of Sampaio et al. (18). However, GLS was load-dependent, and the increase of afterload will underestimate the true value of myocardial strain, which might affect the accuracy of its evaluation of cardiac function. Compared with GLS, non-invasive PSL technology can evaluate cardiac systolic function more objectively and accurately and reflect myocardial oxygen consumption by comprehensively considering myocardial deformation and afterload (19). At present, this technique has made significant progress in the diagnosis of cardiovascular diseases, such as hypertension, dilated cardiomyopathy, myocardial amyloidosis, and coronary artery diseases with preserved ejection fraction, and its feasibility and application value have been confirmed (20–22).

The results of this study showed that GWI, GCW, GWE and GWW in Child-Pugh B and C groups were significantly different from controls, but there was no significant difference in LVEF among all groups. This indicates that myocardial work parameters may reflect subclinical myocardial function damage. Impaired liver function and portal hypertension in patients with cirrhosis can lead to increase vasodilator substances in the body, which may result in peripheral vascular dilatation and reduced afterload (23). In addition, the blood volume was redistributed and the circulation was in a hyperdynamic state in patients with cirrhosis, which masked the performance of reduced left ventricular systolic function, resulting in no significant change in LVEF (24, 25). In addition, compared with Child-Pugh A and B groups, the GWI, GCW and GWE in Child-Pugh C group were further decreased, while the GWW was further increased, indicating that the left ventricular systolic function of patients with cirrhosis decreased gradually with the deterioration of liver function. This may be related to impaired function of β-receptors on the surface of myocardial membrane, altered transmembrane currents and overproduction of cardiodepressant factors such as nitric oxide, endocannabinoid and cytokines, which can inhibite the contraction of myocardial cells (26). These reasons may lead to the decrease of the myocardial work index and effective work, manifesting as the decrease of GWI, GCW and GWE. Meanwhile, the electrical signal conduction of myocardium in patients with cirrhosis is interrupted or delayed due to myocardial fibrosis, which may lead to asynchronous myocardial contraction, as shown by the elongation of myocardial cells during systole phase, resulting in reduced GCW and increased GWW (27). In addition, the correlation analysis results showed that GWI, GCW and GWE were negatively correlated with Child-Pugh classification, while GWW was positively correlated with Child-Pugh classification. This also indicates that with the aggravation of cirrhosis, the left ventricular systolic function decreased gradually. GWW is on the rise, and GWI, GCW and GWE are on the decline.

Furthermore, this study showed that ALB and GLS were independent predictors for GWE in patients with cirrhosis. The decrease of plasma colloid osmotic pressure and insufficient effective blood volume in patients with cirrhosis can activate the sympathetic nervous system and the renin-angiotensin-aldosterone system, resulting in a compensatory state of hyperkinetic circulation with high cardiac output and low peripheral resistance (28). Patients with lower ALB levels may be in a state of greater cardiac volume overload. GLS mainly reflects the longitudinal strain of myocardium in the subendocardial region, which is prone to ischemia and fibrosis (29). The higher cardiac capacity load and greater stress on the subendocardial myocardium in patients with cirrhosis may be prone to microvascular dysfunction, fibrosis and other changes resulting in systolic dysfunction. CCM consortium recommended that absolute values of GLS can be used to detect left ventricular systolic dysfunction in cirrhotic patients with preserved LVEF (30). Recent studies have shown that GLS is also of great significance in predicting poor prognosis and risk stratification in patients with heart failure (31, 32).

## 5. Limitations

Several limitations of the present study should be acknowledged. Firstly, this is a retrospective, single-center study with a small sample size and needs to be expanded for further study to confirm our results. Secondly, most patients with hepatitis B cirrhosis included in this study had taken drugs to control disease progression, and the effects of drugs on left ventricular function cannot be ruled out. Thirdly, we only evaluated the global myocardial function of left ventricle, and the regional myocardial work of 17 segments was not assessed. Finally, we only included patients with cirrhosis caused by hepatitis B virus, and excluded patients with alcoholic cirrhosis or other causes, which may limit the generalizability of our findings in clinical applications.

## 6. Conclusions

The non-invasive LVPSL technology can quantitatively analyze the changes of left ventricular myocardial function in patients with hepatitis B cirrhosis under different disease states. The myocardial work parameters of left ventricular were significantly correlated with liver function classification. GWE was independently correlated with ALB and GLS respectively. This technique can sensitively detect abnormal cardiac function in cirrhosis patients with preserved LVEF, which has a broad prospect of clinical application.

## Data Availability

The original contributions presented in the study are included in the article/Supplementary Material, further inquiries can be directed to the corresponding author/s.
